# Characteristics of Polycyclic Aromatic Hydrocarbons (PAHs) and Common Air Pollutants at Wajima, a Remote Background Site in Japan

**DOI:** 10.3390/ijerph17030957

**Published:** 2020-02-04

**Authors:** Xuan Zhang, Lulu Zhang, Lu Yang, Quanyu Zhou, Wanli Xing, Akira Toriba, Kazuichi Hayakawa, Yongjie Wei, Ning Tang

**Affiliations:** 1 Graduate School of Medical Sciences, Kanazawa University, Kakuma-machi, Kanazawa 920-1192, Japan; zhangxuan@stu.kanazawa-u.ac.jp (X.Z.); zhang-lulu@stu.kanazawa-u.ac.jp (L.Z.); veronicayl@stu.kanazawa-u.ac.jp (L.Y.); zhouquanyu@stu.kanazawa-u.ac.jp (Q.Z.); xingwanli@stu.kanazawa-u.ac.jp (W.X.); 2 Institute of Medical, Pharmaceutical and Health Sciences, Kanazawa University, Kakuma-machi, Kanazawa 920-1192, Japan; toriba@p.kanazawa-u.ac.jp; 3 Institute of Nature and Environmental Technology, Kanazawa University, Kakuma- machi, Kanazawa 920-1192, Japan; hayakawa@p.kanazawa-u.ac.jp; 4 State Key Laboratory of Environmental Criteria and Risk Assessment, Chinese Research Academy of Environment Sciences, Beijing 100012, China

**Keywords:** air pollution, long-range transport, trace gas, ozone, PM_2.5_

## Abstract

*Background*: Background sites are mainly affected by long-range-transported air pollutants, resulting in potential adverse effects on local atmospheric environments. A 4–5 year observational study was conducted to illustrate the air pollution profile at the Kanazawa University Wajima air monitoring station (KUWAMS), an ideal remote background site in Japan. *Methods*: Nine polycyclic aromatic hydrocarbons (PAHs) in the particulate phase and various air pollutants were continuously monitored for 4–5 years. Diagnostic ratios of PAHs and back-trajectory analysis were applied to trace the possible sources of the air pollutants collected at the sampling site. *Results*: The atmospheric concentration of PAHs in the atmosphere at the site decreased from 2014 to 2019, benefit from the predominant air pollution control policy in China and Japan. Common air pollutants including sulfur dioxide (SO_2_), nitrogen oxides (NO_x_), ozone, methane (CH_4_), and non-methane hydrocarbon (NMHC) were detected in low concentrations from 2016 to 2019, while ozone (O_3_) and particulate matter (PM_2.5_, PM with a diameter less than 2.5 μm) were present in high levels that exceeded the Japanese standards. Most air pollutants peaked in spring and showed evident diurnal variations in spring and summer. *Conclusions*: This is the first study to clarify the atmospheric behaviors of multiple air pollutants at a background site in Japan. Significant external air pollutant impact and unneglectable air pollution were demonstrated at KUWAMS, indicating the importance of studying atmospheric pollution at remote sites.

## 1. Introduction

As a global problem, air pollution causes adverse effects in many parts of the world [1,2,3,4,5]. According to the World Health Organization (WHO), approximately 90% of the world’s population lives in locations with severely polluted air [6].

Particulate matter (PM), ground-level ozone (O_3_), sulfur dioxide (SO_2_), and nitrogen dioxide (NO_2_) are listed as criteria pollutants for determination of the air quality indexes of many countries and organizations, such as WHO, China, United States, and the European Union. Due to their wide dispersion and great human health risk, many epidemiological researches have been conducted to clarify the exposure–response relationship [7,8,9,10]. PM consists of solid and liquid particles suspended in the air and represents the sum of a variety of complex pollutants. Polycyclic aromatic hydrocarbons (PAHs), as components of PM, are a group of persistent organic pollutants with two or more fused benzenoid rings. Some PAHs are well known for their carcinogenicity and mutagenicity and therefore pose a serious threat to human health [11,12,13]. SO_2_ is mainly emitted from sulfur-containing fossil fuel combustion. Once produced, SO_2_ is easily oxidized to sulfuric acid or sulfate aerosols, which can induce acid deposition. Exposure to SO_2_ affects the respiratory system [10,14] and causes irritation of the eyes [15]. Nitrogen oxides (NO_x_: NO_2_ and NO) originate from primary sources like fuel burning. NO_x_ plays important roles in the formation of photochemical smog (precursor of O_3_) and acid rain. Exposure to NO_2_ can increase symptoms of bronchitis and asthma, as well as lead to respiratory infections and reduced lung function and growth [10]. Stratospheric O_3_ can protect humans from ultraviolet exposure, but elevated tropospheric O_3_ is a major and active photochemical oxidant that is one of the predominant components of photochemical smog and a key pollutant linked to breathing problems, asthma, reduced lung function, and respiratory diseases [10,16]. Methane (CH_4_) is a vital greenhouse gas, and non-methane hydrocarbons (NMHC) are significant precursors of O_3_.

Generally, urban areas suffer mostly from air pollution due to the presence of extensive anthropogenic sources, such as industrial activity [9,17] and vehicle emissions [18]. However, studies and observations have shown that air pollutants derived from heavy-polluted areas have unignorable effects on remote background sites through regional or continental transport from polluted sites [19,20,21,22,23]. The influence of transported pollutants is subject to several factors, such as potential chemical reactions during transport or at the recipient location [24,25,26], which complicate the effects and cause new potential adverse effects on the atmospheric environment of background areas. Local meteorological conditions can also impact the atmospheric behaviors of transported air pollutants [21]. Therefore, it is important to understand the air pollution profile in remote areas acting as a receptor of transported air pollutants to comprehensively evaluate the health effects of air pollution.

According to different atmospheric lifetimes, air pollutants can be regionally or continentally transported in air masses. Based on our previous research, PAHs detected at an ideal background site, Kanazawa University Wajima Air Monitoring Station (KUWAMS), were transported from northeastern China in the cold season and domestic Japan in the warm season and exhibited certain variations over time [19,21,27,28]. Additionally, it is of interest to determine the atmospheric behaviors of other air pollutants and illustrate the evolution of air masses from the Asian continent to Japan and the contribution of domestic emission sources to air pollution at the background site. Therefore, in the present study, long-term observation of several air pollutants was carried out to obtain a comprehensive understanding of the air pollution at a typical background location. To our knowledge, this is the first exhaustive study involving a detailed air pollutant profile at a background site in Japan.

## 2. Materials and Methods

### 2.1. Sampling Site

**Figure 1:**
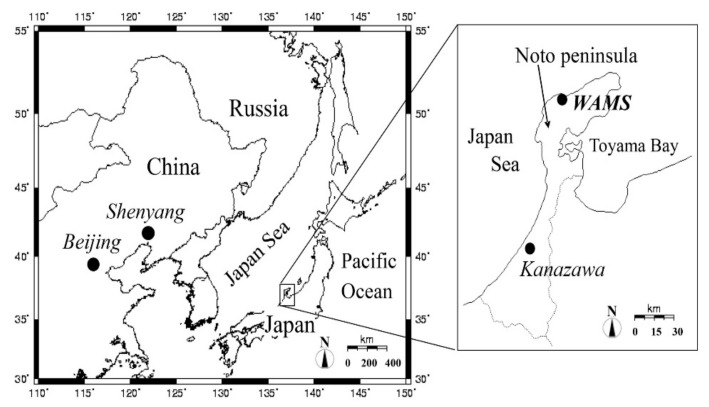
Location of the sampling site. (Kanazawa University Wajima Air Monitoring Station (KUWAMS): 37.4°N, 136.9°E; 60 m above sea level [27]).

Long-term observation for common air pollutants and meteorological parameters was conducted at KUWAMS (Figure 1, Nishifutamata-machi, Wajima City, Ishikawa Prefecture, Japan, 37.4 °N, 136.9 °E; 60 m above sea level). Wajima is situated in the northern Ishikawa Prefecture, Japan, with a population of 26,582 as of 2019/11. Tourism, lacquerware, and fishery are the main local industries [29].

KUWAMS is encompassed by mountains with an extensive forest and is 2.1 km south of the Sea of Japan coast. No major industrial emissions occur around this site. Air masses from the Asian continent can pass through KUWAMS in the cold season and domestic Japan in the warm season. Benefitting from its unique location, KUWAMS is a distinctive background air monitoring station for transboundary atmospheric pollutant studies in Japan. In our previous research [19,21,27,28], PAHs collected at KUWAMS were long-range-transported from northeastern China in winter and domestic Japan in summer.

### 2.2. Total Suspended Particulate (TSP) Total Sampling and PAH Measurement

TSPs were collected by a high-volume air sampler (AH-600, Sibata Sci. Tech. Ltd., Saitama, Japan) with a quartz fiber filter (8 inch × 10 inch, 2500QAT- UP, Pallflex Products, Putnam, CT, USA) from 2014/6 to 2019/8. The flow rate was 700 L min ^−1^. Filters were changed every seven days. After drying in a desiccator in the dark, the filters were weighed and then stored at −20 °C until PAH analysis. 

A total of nine PAHs (4 to 6 rings) were detected in TSP: fluoranthene (Flu), pyrene (Pyr), benz[*a*]anthracene (BaA), chrysene (Chr), benzo[*b*]fluoranthene (BbF), benzo[*k*]fluoranthene (BkF), benzo[*a*]pyrene, benzo[*ghi*]perylene (BgPe), and indeno [1,2,3-*cd*]pyrene (IDP). After pretreatment, high-performance liquid chromatography with fluorescence detection was used to measure the PAH concentrations. Detailed procedures can be found in our previous study [21]. A mixture of PAHs (Untied State Environmental Protection Agency (USEPA) 610 PAH mix) was purchased from Supelco Park (Bellefonte, PA, USA). Two internal standards for PAHs (pyrene-*d*10 (Pyr-*d*10) and benzo[*a*] pyrene-*d*12 (BaP-*d*12)) were purchased from Wako Pure Chemicals (Osaka, Japan). All other chemicals used were of analytical reagent grade.

### 2.3. Online Monitoring of Air Pollutants

Air pollutants including PM_1_, PM_2.5_, NO_x_, SO_2,_ O_3_, and total hydrocarbons (THC: CH_4_ and NMHC) and the organic carbon (OC) and elemental carbon (EC) in PM_2.5_ were monitored online from 2014/4 to 2019/8 at KUWAMS. In detail, PM_1_ and PM_2.5_ were measured by particulate matter monitor PM-714 in accordance with the Beta eta ray attenuation method with a flow rate of 16.7 L/min. NO_X_, SO_2_, and O_3_ were analyzed at a flow rate of 1 L/min with nitrogen oxides analyzer NA-721 (chemiluminescence method), sulfur dioxide analyzer SA-731 (ultraviolet fluorescence method), and ozone analyzer OA-781 (nondispersive ultraviolet absorption method), respectively. THC were monitored with hydrocarbon analyzer HA-771 according to the hydrogen flame ionization detection method at a flow rate of 0.5 L/min. In addition, the OC and EC in PM_2.5_ were measured using automatic particulate carbon monitor APC-710 based on the near-infrared/UV absorption method at a flow rate of 16.7 L/min. Meteorological conditions were simultaneously monitored by the weather Transmitter WXT530 and included temperature (T), relative humidity (RH), pressure (P), wind speed (WS), wind direction (WD), rainfall (rain), and hail. Data per minute, hour, and day were recorded automatically and downloaded from the samplers every month. The detection limit for NO_X_, SO_2_, and O_3_ was 1 ppb. All auto-monitoring instruments were purchased from Kimoto Electric Co. Ltd (Osaka, Japan). In addition, sunshine duration data were downloaded from the Japan Meteorological Agency as supplementary information [30].

### 2.4. Quality Control

Blank filters were processed at the sampling site together with the samples. Three field blank filters were selected randomly from filters with the same lot number. No contamination from filter transport was detected. Before detecting PAHs, standard solutions of each PAH were injected into systems for method validation. The correlation coefficients for the calibration curves of all PAHs were greater than 0.997. The average recovery of internal standards and each PAH was 100% ± 20%. The detection limit of each PAH was in the range of 0.3–1.3 ng/mL.

The continuous monitoring instruments were checked monthly and exhaustively examined by professionals every year. Outliers resulting from regular maintenance activities (e.g., periodic zero-span checks, calibrations, etc.) and abnormal disruptions were excluded from analysis.

### 2.5. Back-Trajectory Analysis

Backward-trajectory analyses were performed using the window-based HYSPLIT 4 (Hybrid Single-Particle Lagrangian Integrated Trajectory) model developed by the American National Oceanic and Atmospheric Administration (NOAA). HYSPLIT was widely applied on air mass tracking [22,28,31,32]. In this study, HYSPLIT was performed to clarify the potential sources of common air pollutants at KUWAMS. Initial height was set as 1000 m. Total run time was set with accordance to atmospheric lifetime of air pollutants.

### 2.6. Statistical Analysis

Hourly data were used to describe the profiles of auto-monitored pollutants and meteorological conditions. Air pollutant concentrations were calculated based on the Manual for Continuous Monitoring of Air Pollution of Japan [33]. The sampling period was divided into warm (June to October) and cold (November to May of the next year) period for PAH analysis due to different sources [21], while four seasons (spring: March to May; summer: June to August; autumn: September to November; winter: December to February of the next year) were used for analysis of the other pollutants based on the distinct seasonal meteorological parameters. Weekly averages were computed for the meteorological parameters and used to perform Spearman correlation analysis to determine their association with PAHs. An independent-samples test was used to compare the differences between annual and seasonal PAH concentrations. A *p*-value of less than 0.05 indicates that the results are statistically significant. Data were evaluated with SPSS 25.0 (IBM Corp., Armonk, New York, USA).

## 3. Results and Discussion

### 3.1. Overview

#### 3.1.1. PAHs

**Figure 2:**
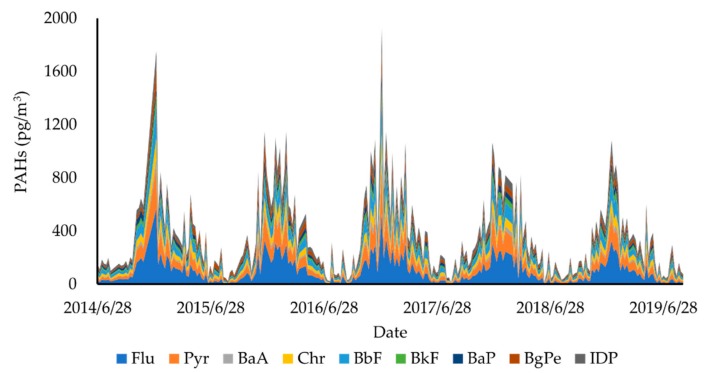
Weekly variation in Polycyclic Aromatic Hydrocarbons (PAHs) from 2014/6 to 2019/8 at Kanazawa University Wajima air monitoring station (KUWAMS).

Figure 2 and Table 1 present the concentration profile of total PAHs in TSP at KUWAMS from 2014/6 to 2019/8. The concentration of total PAHs ranged from 11 to 1923 pg/m^3^ with a mean value of 364 pg/m^3^ during the sampling period. The average concentrations were 143 and 548 pg/m^3^ in the warm and cold seasons, respectively. Individual PAH concentration widely ranged from 0 to 566 pg/m^3^ with an ascending order of Flu > Pyr > BbF > Chr > IDP > BgPe > BaP > BkF > BaA. Compared with past data on these nine PAHs at KUWAMS, the concentration of total PAHs in the TSP observed in this study was lower than that observed from 2004 to 2014 (670 pg/m^3^ in the cold period and 170 pg/m^3^ in the warm period).

In a general comparison with other background sites, the total PAH concentration at KUWAMS was within the ranges detected at urban background sites in Thessaloniki, Greece [34] (5.8 ± 8.4 and 5.9 ± 8.5 ng/m^3^ for Σ13 PAHs bound to PM_10_ and PM_2.5_, respectively); Dongying, China [35] (18.95 ± 16.51 ng/m^3^ for Σ15 PAHs bound to PM_18_); Gosan, Korea [36] (2.9 ng m^−3^ for ∑14 PAHs bound to PM_2.5_, detected in a study on gaseous and particulate PAHs at the Gosan background site in East Asia); and Virolahti, Finland [37] (4.07 ng/m^3^ for ∑11 PAHs bound to PM_10_).

#### 3.1.2. Continuously Monitored Pollutants and Meteorological Conditions

Table S1 presents the meteorological parameters at KUWAMS. Spring was characterized by low temperature, relative humidity and pressure, as well as strong solar radiation. Summer was similar to spring but with the highest temperature and lowest pressure. Autumn had the same high relative humidity as summer. Wind speed and air pressure were highest in winter. Generally, all the weather conditions except for air pressure presented the same daily diurnal changes in all seasons. Spring was characterized by the most evident diurnal weather changes. Summer was only slightly weaker than spring but with a less dramatic daytime decrease in relative humidity. Therefore, atmospheric activity (such as oxidation and photolysis) was most active in spring because of the favorable meteorological conditions, followed by summer, and was rather stable in autumn and winter.

**Figure 3:**
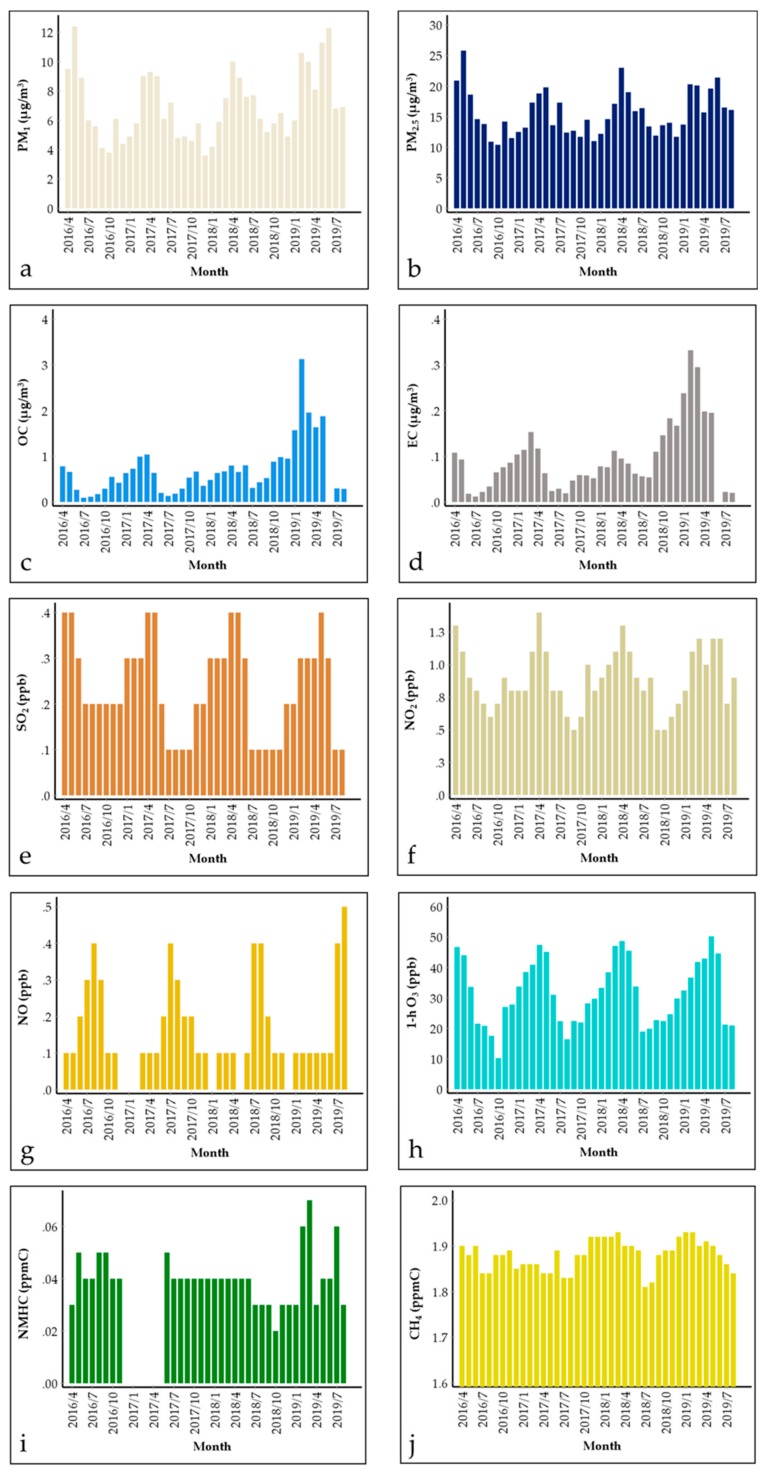
Monthly variation in continuously monitored pollutants from 2014/6 to 2019/8 at Kanazawa University Wajima air monitoring station (KUWAMS). (**a**) PM_1_ (μg/m^3^); (**b**) PM_2.5_ (μg/m^3^); (**c**) OC (μg/m^3^); (**d**) EC (μg/m^3^); (**e**) SO_2_ (ppb); (**f**) NO_2_ (ppb); (**g**) NO (ppb); (**h**) 1-h O_3_ (ppb); (**i**) NMHC (ppmC); (**j**) CH_4_ (ppmC).

Figure 3 and Table S1 represent the concentration profiles of the continuously monitored air pollutants. The daily average concentrations of PM_1_ and PM_2.5_ (Figure 3a,b) were 7.1 ± 5.2 and 15.9 ± 9.5 μg/m^3^, respectively. The annual concentrations were 6.7, 6.0, and 7.4 μg/m^3^ for PM_1_ and 15.4, 14.5, and 16.0 μg/m^3^ for PM_2.5_ in 2016, 2017, and 2018, respectively. Notably, the PM_2.5_ concentration at KUWAMS was higher than the annual average concentration in Japan [38] (11.9 and 11.6 μg/m^3^ in 2016 and 2017, respectively; data from the general air monitoring station, same data source below), even exceeding the annual standard for Japan (15 μg/m^3^). Furthermore, a daily mean PM_2.5_ concentration exceeding the Japan standard (35 μg/m^3^) occurred on 17 days, most of which were in spring (6 days in April, 8 days in May, and 1 day each in February, March, and July). The concentrations of OC and EC in PM_2.5_ (Figure 3c,d) were 0.987 ± 1.341 and 0.146 ± 0.165 μg/m^3^, respectively, which were obviously lower than those detected in PM_2.5_ at a rural site in China [39] (3.53 and 1.05 μg/m^3^). The annual concentrations were 0.504, 0.506, and 1.128 μg/m^3^ for OC and 0.081, 0.069, and 0.170 μg/m^3^ for EC in 2016, 2017, and 2018, respectively.

The average concentrations of SO_2_, NO_2_, and NO (Figure 3e–g) were 0.0–9.9, 0.1–6.7, and 0.0–6.8 ppb, with average values of 0.2 ± 0.3, 0.9 ± 0.5, and 0.1 ± 0.3 ppb, respectively. Data per minute of 98%, 65%, 97% were lower than the detection limit for SO_2_, NO_2_, and NO, respectively. During the sampling period, the atmospheric levels of SO_2_, NO_2_, and NO were almost ten times lower than the average levels in Japan in 2016 and 2017 (9 ppb NO_2_, 2 (2017)-3 (2016) ppb NO, 2 ppb SO_2_). The daily 1 h max average O_3_ concentration (Figure 3h) was 43.6 ± 16.2 (15.3–105.9) ppb, and the daily 16 h average O_3_ concentration (from 5:00 to 20:00) was 31.5 ppb. Notably, there were 12% days with a 1 h daytime value higher than the Japanese standard (60 ppb), indicating a significant atmospheric concentration of O_3_ at KUWAMS. In addition, the annual concentration was 39.2 ± 12.9, 44.8 ± 14.0, and 43.6 ± 14.8 μg/m^3^ for the daily 1 h max average and 28.5 ± 14.8, 31.7 ± 17.0, and 30.5 ± 16.6 μg/m^3^ for the 16 h average in 2016, 2017, and 2018, which were lower than the average level in Japan in 2016 and 2017. NMHC and CH_4_ (Figure 3i,j) showed stable concentrations during the sampling period, with average concentrations of 0.04 ± 0.04 and 1.88 ± 0.04 ppmC, respectively. As a precursor of O_3_, the 3 h (from 6 to 9 a.m.) average NMHC standard is set to 0.2–0.31 ppmC as a reference value for the prediction of the daytime O_3_ concentration in Japan. The 3 h mean concentration of NMHC was 0.04 ± 0.04 ppmC at KUWAMS, which was lower than the average value in Japan in 2016 and 2017 (0.12 ppmC in both years) and the Japanese standard.

The atmospheric concentrations of common air pollutants at KUWAMS, as a remote site, were basically at background levels. However, PM_2.5_ and O_3_ (listed in six pollutant criteria in Japan) were detected at high concentrations during the observation period, which highlights the importance of clarifying their sources, variations, and impacting factors.

### 3.2. Annual, Seasonal and Diurnal Variations

#### 3.2.1. Yearly Variations in PAHs

In general, total PAHs decreased 13% in the cold season (from 527 pg/m^3^ in 2014 to 456 pg/m^3^ in 2018) and 14% in the warm season (from 154 pg/m^3^ in 2014 to 132 pg/m^3^ in 2019). Obvious variation was observed during the 5 years study, with a more pronounced annual divergence in the cold season (*p* = 0.060–0.341) than in the warm season (*p* = 0.108–0.997). In detail, in the cold period, PAH concentrations showed a single peak pattern: the concentration of total PAHs increased from 527 pg/m^3^ (2014) to 534 pg/m^3^ (2015) and peaked at 609 pg/m^3^ in 2016. Then, the PAH concentration decreased to 509 pg/m^3^ in 2017 and to 456 pg/m^3^ in 2018. On the other hand, relatively small wavy fluctuations occurred in the warm period. The average concentrations were 154, 161, 145, 154, 107, and 132 pg/m^3^ in each year from 2014 to 2019.

Based on our previous studies [19,21,27,28], PAHs were mainly transported from the Asian continent in the cold season and domestic Japan in the warm season. For instance, preparation for Beijing Olympic Games in China caused an evident change during cold season from 2006 to 2009, while reconstruction after Noto peninsula earthquake led to a gradual increase from 2007 to 2010 during warm season [21]. In the recent years, PAH concentration revealed a descending trend in the cold and warm season, which might benefit from the air pollution control measures in both regions [38,40]. In addition, the annual variation in total PAHs was caused by the simultaneous fluctuation of 4- to 6-ring PAHs in the cold season, along with a synchronous change in the total PAH/TSP ratio (not shown here), suggesting the evident impact of Asian Continent flow. However, the simultaneous changes in total PAHs, 4- to 6-ring PAHs, and the total PAH/TSP ratio were not seen in the warm season due to more mixed sources (domestic Japan and local).

#### 3.2.2. Seasonal and Diurnal Variations in the Continuously Monitored Pollutants

Figure 3, Figure S1, and Table S2 display the seasonal and diurnal variations in the auto-monitored pollutants. PM_1_ and PM_2.5_ (Figure 3a,b) concentrations had the same seasonality: spring > summer > winter, autumn. It is thought that natural sources such as seasonal pollen and sea salt dominantly contribute to the PM variation at KUWAMS due to its surrounding environment. Additionally, Asian dust is prevailing in spring and can be long-range-transported to Japan, contributed to higher PM level in spring at KUWAMS [41,42]. Besides, distinct peaks in autumn (especially November) were displayed in PM_1_ and PM_2.5_, suggesting the probable contribution of local agricultural residue burning. Winter is the “clean” season due to lack of primary emission and weak atmospheric reactions. Daily PM_2.5_ variation (Figure S1b) reached a valley at approximately 9:00 and peaked at 18:00, and decreased steadily at night. PM_1_ was associated with PM_2.5_ on the season level but with a more solar radiation dependence in diurnal variation. The ratio of PM_1_/PM_2.5_ was 44%, which was lower than that of the Gosan ABC superstation in Korea [43] (60.8%, August 2017–September 2018) and Tuoji Island in China [44] (72%, winter 2014). Nevertheless, PM_1_ variation (Figure S1a) displayed no valley in the late morning but a moderately broad peak (narrower in autumn and winter) from 8:00 to 20:00. Increasing daytime concentrations indicated secondary aerosol formation of PM_1_ and PM_2.5_, while the difference in the daily PM_1_ and PM_2.5_ variations was probably caused by the diversity in the components resulting from the different particle diameters [45,46]. Water-soluble inorganic ions (WSII) are a significant component of PM [47], especially in the coastal area. It was observed that secondary WSII displayed size-fractioned characteristics as significantly abundant in PM_1_ [43,44], which probably led to PM_1_ increase in the daytime. OC and EC (Figure 3c,d) also peaked in spring (winter > autumn > summer). The daytime variation (Figure S1c,d) was different from that of PM_2.5_, and similar fluctuations were observed in only OC and PM_1_. The OC/EC ratio over 2 indicates formation of secondary organic carbon (SOC) [48]. In the current study, the mean value of OC/EC was 6.8.

SO_2_, NO_2_, and O_3_ displayed similar seasonal variations (Figure 3e,f,h), spring > winter > summer > autumn, and diurnal variations (Figure S1e,f,h), daytime > night, indicating the important impact of external inputs. Spring peak of O_3_ commonly occurs at Northern hemisphere, which was explained by various mechanisms [49]. It is thought that air pollutants including O_3_ precursors were long-range transported from continental flow in cold season (winter and spring), and the formation of O_3_ was strengthened by the mounting solar radiation from winter to spring. Low concentration in summer and autumn can be explained by lack of O_3_ precursors and strengthened wash-out effect due to frequent precipitation. Daily O_3_ concentration was dependent on solar radiation intensity. Furthermore, two peaks were detected in the daytime NO_2_ concentration in summer. NO_2_ first peaked in value at 8:00, reached a valley at 14:00, and then peaked again at 18:00. It is thought that NO_2_ photolyzed to produce O_3_ in the daytime in summer.

The NO concentration (Figure 3g, Figure S1g) was extremely low at KUWAMS because of scarce anthropogenic emissions in the area [19,21,27,28]. Interestingly, the NO concentration was highest in summer, resulting from vehicle emission due to increasing tourists. Besides, a morning peak occurred at approximately 8:00 in each season, which was most evident in spring and summer (occurred at 10:00 in winter), suggesting a contribution from passing vehicles, since KUWAMS is located near a shortcut from Anamizuvillage to Wajima City, despite the light traffic.

CH_4_ (Figure 3i, Figure S1i) presented a seasonal variation of winter > spring > autumn > summer but no evident daily variation. Seasonal features of CH_4_ in a background environment are correlated with its atmospheric reaction with hydroxyl radical, which depends on the seasonal intensity of ultraviolet radiation [50]. No obvious seasonal trend was displayed in NMHC (Figure 3j). In spring and summer, the concentration of NMHC reached a daily high at 14:00 (Figure S1j). KUWAMS is encompassed with abundant forest, which can produce considerable isoprene. Thus, the daily high was probably due to isoprene variation [51]. Nevertheless, it should be further explored since only total NMHC concentrations were available in this study.

In general, significant atmospheric concentration levels of common air pollutants were primarily observed in spring. Diurnal concentration changes were most evident in spring and summer at KUWAMS.

### 3.3. Meteorological Conditions

Atmospheric levels of PAHs can be influenced by meteorological parameters [21,27]. Spearman correlation was conducted to clarify the correlations of PAH concentrations with meteorological conditions. As shown in Table 2, a negative relationship was found between temperature and all PAHs at KUWAMS, which was stronger in the cold season and for the 4-ring PAHs. The physicochemical properties of PAHs can affect the phase in which they reside. As the temperature increases, 4-ring PAHs with high vapor pressure are more prone to volatilize into the gas phase from the particulate phase than PAHs with more rings [52,53]. In contrast, pressure was positively associated with the concentrations of each PAH, especially those of 4-ring PAHs, which can be explained by the negative relation between pressure and temperature [21]. Additionally, wind speed increased PAH levels in the cold season. Generally, wind can accelerate air flow, which affects the process by which local air pollutants spread to downwind sites or are transported from distant locations. A positive correlation indicated that a surrounding air mass containing PAHs was transported to KUWAMS. Finally, no significant correlations were found between the relative humidity and any of the PAHs.

### 3.4. Source Apportionment

#### 3.4.1. Diagnostic Ratios (DRs) of PAHs

Atmospheric PAHs primarily originate from the incomplete combustion and pyrolysis of organic matter [33], and different sources differ in the dominant PAHs and/or the proportions of specific PAHs [34,35]. The type of PAHs produced strongly depends on the combustion temperature [54,55]. PAHs with low molecular weight are generated via low-temperature burning, whereas high-molecular-weight PAHs account for a larger proportion with increasing temperature. The DRs of specific PAHs are extensively used to infer PAH sources [12,19,21,22,28,56]. Typical DRs are listed in Table 3. Taking the ratio of Flu/(Flu + Pyr) as an example, a value below 0.4 indicates unburned petroleum as the main PAH source, a value from 0.4 to 0.5 suggests a liquid fossil fuel source, and a value above 0.5 implies that wood and coal combustion make a large contribution to PAHs. In this study, the mean concentration ratios of Flu/(Flu/Pyr), BaA/(BaA + Chr), IDP/(IDP + BgPe), BbF/(BbF + BkF), and BaP/BgPe in the cold (warm) season were 0.53–0.68 (0.24–0.66), 0.19–0.40 (0.16–0.60), 0.20–0.72 (0.15–0.65), 0.70–0.76 (0.70–0.87), and 0.22–2.31 (0.20–1.84), respectively, suggesting that PAHs were mainly from mixed sources including coal and biomass combustion and vehicle emission. Moreover, BaA/(BaA + Chr) was also used to identify fresh and aged air masses. Relatively low BaA/(BaA + Chr) ratios were found in both the warm and cold periods, suggesting a predominant proportion of PAHs that had undergone reaction or long-range transport, instead of PAHs from local emissions, at KUWAMS, which was consistent with our previous report [21,22,27]. However, factors such as complicated atmospheric behavior can result in spatiotemporal changes in atmospheric PAHs. Thus, DRs should be used with prudence or primarily to study PAH fingerprints.

#### 3.4.2. Back-Trajectory Analysis

**Figure 4:**
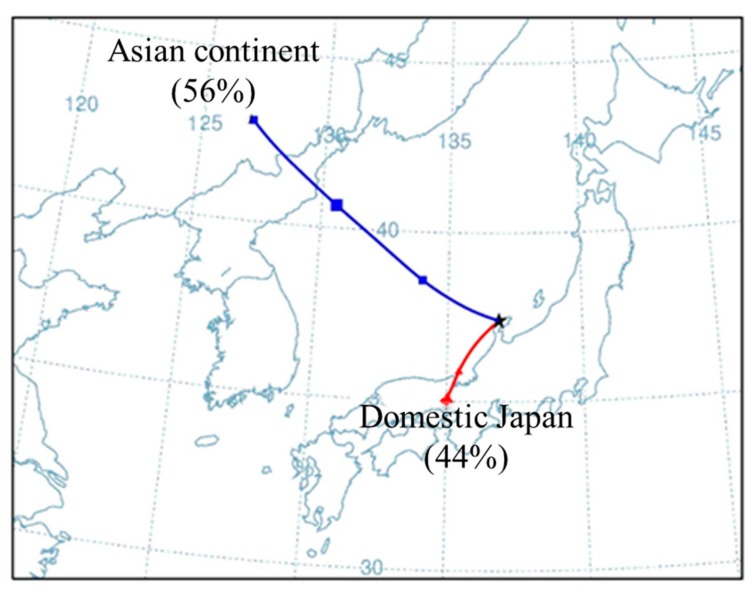
Back-trajectory analysis in spring from 2016/4 to 2019/5 at Kanazawa University Wajima air monitoring station (KUWAMS) (37.4°N, 136.9°E). Starting time: every hour from 11:00 to 15:00; Starting height: 1000 m above ground level.

Based on the above results, spring was the most “polluted” seasons, with high external air pollutant input. Thus, back-trajectory analysis was performed to trace the possible source of air pollutants in spring at KUWAMS from 2016/4 to 2019/5. The starting height was set to 1000 m above ground level, and the starting time was set at each hour from 11:00 to 15:00 because the concentration peak of most pollutants was found to fall in this time interval. Trajectories of air masses were tracked every day and clustered into appropriate numbers of groups for further analysis. The final result is shown in Figure 4.

As shown in Figure 4, the air masses transported to KUWAMS were mainly from the Asian continent and domestic Japan in nearly comparable contributions. In detail, 56% of the air masses derived from the Asian continent (northern China) passed over North Korea and the Pacific Ocean and arrived at KUWAMS. The other source was the Kansai Region (Osaka et al.) of Japan, from which air masses passed over the Sea of Japan before finally reaching the sampling site. China is a dominant source of Asian air pollution due to its fast-developing economy. Adjacent locations and favorable meteorological conditions enable the long-range transport of pollutants via air masses. However, high O_3_ concentrations also occur in Japanese metropolitan areas, such as Osaka [38], due to considerable vehicle emissions, especially in spring. Nevertheless, the atmospheric lifetimes of SO_2_, NO_2_, and O_3_ are on order of days, and these species can actively take part in atmospheric reactions. It is unlikely that these short-lived pollutants have direct impacts on remote sites. However, it is thought that short-lived pollutants can be transported to distant locations in different forms. For example, peroxyacetyl nitrates (PAN) are important photooxidants and are generated from only anthropogenic sources by the reaction of NO_x_ and acetaldehyde. PAN can be removed via thermal decomposition reactions and yield NO_2_. Due to its temperature sensitivity, PAN can be transported to remote sites at low temperature and release NO_2_ upon decomposition at increased temperature at the remote site [26]. Sadanaga et al. [36] concluded that total PAN and total organic nitrates undergo long-range transport from the Asian continent to the Noto Peninsula. Thus, it is speculated that these were possibly originated from aged air masses by long-range transport from northern China and from freshly polluted plumes from domestic Japan. Nevertheless, this speculation needs to be further confirmed due to lack of other pollutant data such as that of PAN. In addition, natural sources, such as dimethyl sulfide oxidation to produce SO_2_ and microbial decomposition to generate SO_2_ and NO_x_, also have an impact on pollutant concentrations at KUWAMS.

## 4. Conclusions

In this study, long-term observation of TSP-bound PAHs (5 years) and multiple gaseous pollutants (4 years) was performed at a typical remote background site. Based on the results, the yearly concentration of PAHs decreased from 2014 to 2019 in the cold and warm period, indicating a reduction trend in China and Japan, which resulted from effective implementation of pollution control measures. Diagnostic ratios suggested that PAHs collected at KUWAMS were generated by mixed sources containing coal and biomass burning, as well as vehicle emission. Meteorological conditions played important parts in PAHs atmospheric activity. On the other hand, common air pollutants were present at relatively low levels but with pronounced seasonal features, and high concentrations of PM_2.5_ and O_3_ that exceeded the Japanese standards were detected. CH_4_ and NMHC displayed steady concentration level during sampling period with a winter high occurred in CH_4_. Gaseous pollutants probably derived from northern China and domestic Japan in spring, while local sources such as pollen, sea salt, vehicle emission, and biomass burning also contributed to atmospheric pollution at KUWAMS.

## Figures and Tables

**Table 1:** Concentration profiles of PAHs (pg/m^3^) and TSP (μg/m^3^) from 2014/6 to 2019/8 at Kanazawa University Wajima air monitoring station (KUWAMS).

	Whole Period (n = 2322)		Warm Period (n = 1053)		Cold Period (n = 1269)	
	Range	Mean ± SD	Range	Mean ± SD	Range	Mean ± SD
Flu	2–566	96 ± 93	2–123	29 ± 22	22–566	152 ± 92
Pyr	2–345	61 ± 56	2–76	21 ± 14	12–345	94 ± 57
BaA	0–82	14 ± 13	0–18	5 ± 3	3–82	21 ± 13
Chr	0–167	33 ± 26	0–49	14 ± 10	8–167	49 ± 25
BbF	1–284	54 ± 43	1–75	23 ± 15	13–284	80 ± 43
BkF	0–102	20 ± 16	0–28	9 ± 6	5–102	29 ± 15
BaP	0–129	26 ± 20	0–37	12 ± 8	7–129	37 ± 19
BgPe	1–161	30 ± 22	1–53	16 ± 10	10–161	41 ± 23
IDP	1–152	31 ± 24	1–45	14 ± 9	8–152	45 ± 24
4-ring	7–1130	204 ± 186	7–258	69 ± 47	47–1130	316 ± 185
5-ring	2–514	100 ± 78	2–135	44 ± 28	25–514	145 ± 76
6-ring	2–279	61 ± 45	2–75	31 ± 17	20–279	87 ± 45
Total PAHs	11–1923	364 ± 307	11–458	143 ± 90	94–1923	548 ± 302
TSP	4–64	18 ± 9	4–242	18 ± 23	4–64	20 ± 11

SD: standard variation; Flu: fluoranthene; Pyr: pyrene; BaA: benz[*a*]anthracene; Chr: chrysene; BbF: benzo[*b*]fluoranthene; BkF: benzo[*k*]fluoranthene; BgPe: benzo[*ghi*]perylene; BaP: benzo[*a*]pyrene; IDP: indeno[1,2,3-*cd*]pyrene; PAHs: Polycyclic Aromatic Hydrocarbons; TSP: Total Suspended Particulate.

**Table 2:** Spearman correlation among PAHs and meteorological condition 2014/6 to 2019/8 in the (a) cold season (n = 94) and (b) warm season (n = 80).

(a)						
Pollutants	T	RH	P	WS	Rain	Sunshine
Flu	−0.707 **	0.190	0.517 **	0.377 **	0.260 *	−0.645
Pyr	−0.684 **	0.179	0.501 **	0.373 **	0.287 **	−0.617 **
BaA	−0.696 **	0.189	0.535 **	0.421 **	0.304 **	−0.634 **
Chr	−0.539 **	0.149	0.422 **	0.278 **	0.236 *	−0.511 **
BbF	−0.590 **	0.189	0.473 **	0.304 **	0.271 **	−0.582 **
BkF	−0.563 **	0.161	0.468 **	0.321 **	0.269 **	−0.552 **
BaP	−0.481 **	0.109	0.427 **	0.333 **	0.292 **	−0.485 **
BgPe	−0.434 **	0.121	0.399 **	0.208 *	0.219 *	−0.446 **
IDP	−0.582 **	0.193	0.471 **	0.312 **	0.278 **	−0.577 **
4-ring	−0.686 **	0.181	0.502 **	0.371 **	0.275 **	−0.628 **
5-ring	−0.559 **	0.166	0.465 **	0.317 **	0.278 **	−0.557 **
6-ring	−0.523 **	0.169	0.443 **	0.260 *	0.251 *	−0.524 **
Total PAHs	−0.634 **	0.169	0.486 **	0.345 **	0.276 **	−0.597 **
**(b)**						
**Pollutants**	**T**	**RH**	**P**	**WS**	**Rain**	**Sunshine**
Flu	−0.521 **	−0.147	0.476 **	0.206	0.004	−0.080
Pyr	−0.442 **	−0.121	0.445 **	0.128	−0.052	−0.067
BaA	−0.362 **	−0.089	0.392 **	0.124	−0.062	−0.006
Chr	−0.310 **	−0.083	0.331 **	0.104	−0.070	0.024
BbF	−0.306 **	−0.053	0.341 **	0.118	−0.037	−0.011
BkF	−0.313 **	−0.069	0.346 **	0.115	−0.048	−0.007
BaP	−0.267 *	−0.120	0.301 **	0.109	−0.106	0.093
BgPe	−0.261 *	−0.107	0.298 **	0.127	−0.053	0.033
IDP	−0.338 **	−0.049	0.335 **	0.078	−0.085	−0.005
4-ring	−0.460 **	−0.118	0.450 **	0.166	−0.019	−0.064
5-ring	−0.304 **	−0.070	0.333 **	0.109	−0.059	0.012
6-ring	−0.310 **	−0.075	0.323 **	0.106	−0.054	0.005
Total PAHs	−0.372 **	−0.093	0.386 **	0.145	−0.038	−0.020

* *p* < 0.05, ** *p* < 0.01. Flu: fluoranthene; Pyr: pyrene; BaA: benz[*a*]anthracene; Chr: chrysene; BbF: benzo[*b*]fluoranthene; BkF: benzo[*k*]fluoranthene; BgPe: benzo[*ghi*]perylene; BaP: benzo[*a*]pyrene; IDP: indeno[1,2,3-*cd*]pyrene; T: temperature; RH: relative humidity; P: pressure; WS: wind speed; PAHs: Polycyclic Aromatic Hydrocarbons.

**Table 3:** Diagnostic ratios (DRs) of Polycyclic Aromatic Hydrocarbons (PAHs).

Diagnostic Ratio	Value Range	Source	Cold Season	Warm Season
Flu/(Flu + Pyr)	<0.4	Unburned petroleum [57,58]	0.53–0.68 (0.61)	0.24–0.66 (0.54)
	>0.4	Petrogenic [57,58]		
	0.4–0.5	Liquid fossil fuel [57,58]		
	<0.5	Gasoline		
	>0.5	Wood and coal combustion [58] Diesel [59]		
BaA/(BaA + Chr)	0.2–0.35	Coal combustion [58,60]	0.19–0.40 (0.29)	0.16–0.60 (0.26)
	>0.35	Vehicular emission [58,60]		
IDP/(IDP + BgPe)	0.18 <0.2	Cars [61] Petrogenic [58,60]	0.20–0.72 (0.52)	0.15–0.65 (0.46)
	0.2–0.5	Petroleum combustion [58,60]		
	0.56	Coal [61]		
	0.62	Wood burning [61]		
	>0.5	Grass, wood and coal combustion [58,60]		
BbF/(BbF + BkF)	0.70–0.76	Coal combustion [22]	0.70–0.76 (0.73)	0.70–0.87 (0.73)
BaP/BgPe	0.5–0.6 <1.0 >1.25	Traffic emission [61] Vehicle [62,63] Brown coal [61]	0.22–2.31 (0.86)	0.20–1.84 (0.73)

## Supplementary Materials

The following are available online at https://www.mdpi.com/1660-4601/17/3/957/s1, Table S1: Concentration profiles of continuously monitored pollutants and meteorological conditions from 2016/4 to 2019/8 at Kanazawa University Wajima air monitoring station (KUWAMS), Table S2: Seasonal concentration profiles (mean ± SD) of continuously monitored pollutants and meteorological conditions from 2016/4 to 2019/8 at Kanazawa University Wajima air monitoring station (KUWAMS); Figure S1: Diurnal variation of continuously monitored pollutants from 2016/4 to 2019/8 at Kanazawa University Wajima air monitoring station (KUWAMS). (blue line: spring, red line: summer, green line: autumn, orange line: winter).
