# Supplementary material for: Characteristics of Polycyclic Aromatic Hydrocarbons (PAHs) and Common Air Pollutants at Wajima, a Remote Background Site in Japan
Source: Int J Environ Res Public Health. 2020 Feb 4;17(3):957. doi: 10.3390/ijerph17030957 (PMC7036938; doi:10.3390/ijerph17030957)
Supplement: Supplementary file 1 [file ijerph-17-00957-s001.pdf]

Supplement Information

Table S1. Concentration profiles of continuously monitored pollutants and meteorological conditions from 2016/4 to 2019/8 at Kanazawa University Wajima air monitoring station (KUWAMS).

Parameter	Valued Hours	Range	Mean ± SD
Air pollutant			
PM_1 (μg/m^3)	22606	0.1–42.1	7.1 ± 5.2
$PM_{2.5}$ (μg/m^3)	22606	0.1–141.8	15.9 ± 9.5
OC (μg/m^3)	19056	0.001–101.948	0.987 ± 1.341
EC (μg/m^3)	19056	0.001–5.589	0.146 ± 0.165
SO_2 (ppb)	27128	0.0–9.9	0.2 ± 0.3
NO_2 (ppb)	28319	0.1–6.7	0.9 ± 0.5
NO (ppb)	28319	0.0–6.8	0.1 ± 0.3
1-h O_3 max value (ppb)	28453	15.3–105.9	43.6 ± 16.2
16-h O_3 average (ppb)	19081	0.7–105.9	31.5 ± 17.2
CH_4 (ppmC)	22377	0.04–3.65	1.88 ± 0.04
NMHC (ppmC)	22376	0.01–5.79	0.4 ± 0.1
Meteorological condition			
T (°C)	26245	0.1–36.5	14.6 ± 8.3
RH (%)	26245	3.3–100.0	78.2 ± 14.6
P (hPa)	26245	969.5–1029.6	1007.7 ± 7.0
WD	26245	0–360	178.6 ± 76.2
WS (m/s)	26245	0.1–4.7	0.9 ± 0.5
Rain (mm)	26245	0–50.5	0.2 ± 1.3
Hail (Hits/cm^2)	26245	0–0.4	0 ± 0

ppb: parts per billion, ppmC: parts per million carbon, SD: standard deviation.

Table S2. Seasonal concentration profiles (mean ± SD) of continuously monitored pollutants and meteorological conditions from 2016/4 to 2019/8 at Kanazawa University Wajima air monitoring station (KUWAMS).

Parameter	Spring	Summer	Autumn	Winter
Air pollutant				
PM_1 (μg/m^3)	10.0 ± 6.1	7.2 ± 5.0	5.5 ± 3.6	5.8 ± 4.1
$PM_{2.5}$ (μg/m^3)	20.3 ± 11.7	16.2 ± 9.4	13.2 ± 6.9	13.7 ± 7.5
OC (μg/m^3)	1.327 ± 1.76	0.435 ± 0.470	0.693 ± 0.610	1.089 ± 1.373
EC (μg/m^3)	0.182 ± 0.195	0.034 ± 0.026	0.113 ± 0.115	0.158 ± 0.172
SO_2 (ppb)	0.4 ± 0.5	0.2 ± 0.2	0.2 ± 0.2	0.3 ± 0.2
NO_2 (ppb)	1.2 ± 0.6	0.8 ± 0.4	0.7 ± 0.4	0.9 ± 0.5
NO (ppb)	0.1 ± 0.1	0.4 ± 0.5	0.1 ± 0.3	0.1 ± 0.0
1-h O_3 max value (ppb)	58.6 ± 15.7	36.1 ± 16.2	35.7 ± 12.0	41.2 ± 6.8
16-h O_3 average (ppb)	45.2 ± 16.8	23.6 ± 15.8	22.8 ± 13.5	33.0 ± 9.9
Meteorological condition				
T (°C)	11.7 ± 5.9	22.5 ± 4.4	15.2 ± 5.6	3.8 ± 3.0
RH (%)	72.1 ± 17.7	82.0 ± 11.9	83.0 ± 11.4	76.3 ± 12.6
P (hPa)	1008.2 ± 6.1	1002.7 ± 4.7	1010.7 ± 6.3	1012.9 ± 6.4
WS (m/s)	1.0 ± 0.5	0.6 ± 0.4	0.8 ± 0.4	1.1 ± 0.5
Rain (mm)	0.2 ± 0.8	0.2 ± 1.6	0.3 ± 1.7	0.2 ± 0.9
Hail (Hits/cm^2)	0 ± 0	0 ± 0	0 ± 0	0 ± 0
Sunshine (h)	0.3 ± 0.4	0.3 ± 0.4	0.3 ± 0.4	0.1 ± 0.2

ppb: parts per billion, ppmC: parts per million carbon.

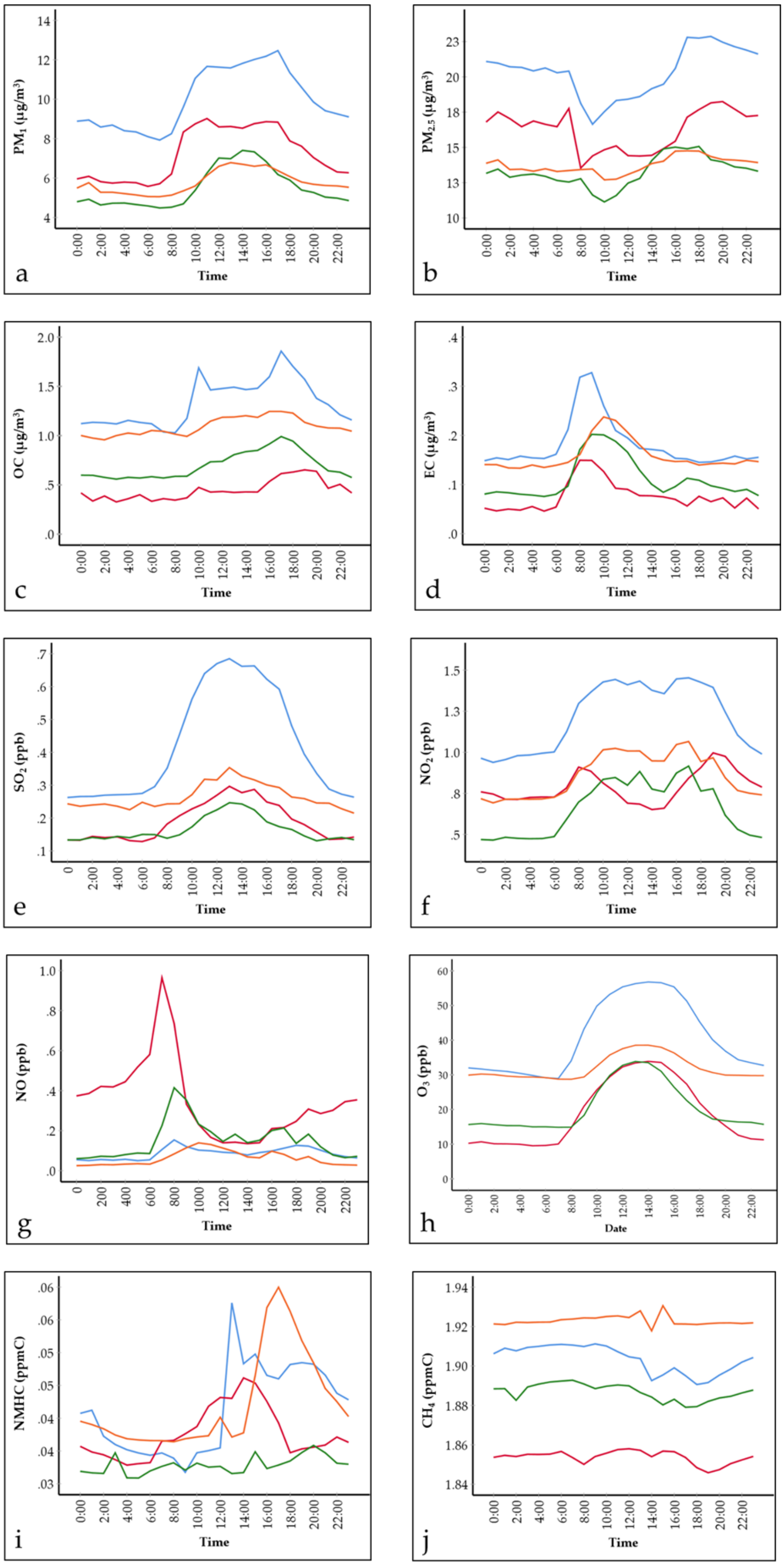

Figure S1. Diurnal variation of continuously monitored pollutants from 2016/4 to 2019/8 at Kanazawa University Wajima air monitoring station (KUWAMS). (blue line: spring, red line: summer, green line: autumn, orange line: winter).
